# Perception of risk of relapse among patients with first episode and recurrent schizophrenia: a descriptive phenomenological study

**DOI:** 10.1186/s12888-023-05023-0

**Published:** 2023-08-10

**Authors:** Hong Yu, Yu-jing Sun, Meng-nan Qin, Jia-xin Ren, Kai Yu, Jin Song, Yu-qiu Zhou, Li Liu

**Affiliations:** Department of Nursing, Harbin Medical University Daqing Campus, Heilongjiang 163319 Daqing, China

**Keywords:** Schizophrenia, Perception, Recurrent risk, Descriptive phenomenology, Qualitative research

## Abstract

**Background:**

Patients suffering from schizophrenia are at a higher risk of relapse. The perception of the risk of relapse in patients is critical for relapse prevention. In the field of psychiatry, the study of risk perception of relapse has been neglected.

**Methods:**

We carried out a qualitative study using a descriptive phenomenological approach. Data were collected at two psychiatric hospitals in China. In total, 22 patients with schizophrenia were recruited through purposive sampling. Face to face semi-structured in-depth interviews were conducted. Interview recordings were transcribed by the research team, and transcripts were analysed by two independent coders with Colaizzi’s descriptive analysis framework. The consolidated criteria for reporting qualitative research checklist were used for reporting.

**Results:**

The data of first-episode patients yielded three themes: (i) lack of knowledge about disease recognition and medical treatment; (ii) overoptimistic estimation of the risk of relapse; (iii) perceived importance of treatment. For first-relapse patients : (i) initial awareness of relapse warning signs; (ii) lack of systematic and accurate assessment of disease information; (iii) the perception that drug withdrawal is related to relapse. Patients with multiple relapses: (i) susceptibility to relapse: confusion and powerlessness; (ii) the severity of relapse: suicidal thoughts and behavior; (iii) effects of perceived benefits and barriers of medication behaviour.

**Conclusions:**

In schizophrenic patients with first-episode, first-relapse, and multiple relapses, there were dynamic changes in the perception of disease relapse risk and medication behaviour. Medical workers must improve risk awareness education. They should provide patients with scientific, accurate, and timely communication channels, and dynamically assess and manage the risk of relapse in various patients.

## Background

Schizophrenia is a chronic, disabling, and severe mental disorder with a high risk of relapse [1]. Although much progress has been made in the prevention and treatment of relapse, there are still challenges [2]. In a retrospective study in which 50 patients were followed for more than 15 years, the relapse rates were 52%, 60%, 86%, and 90% at 2, 5, 10, and 15 years, respectively, after the remission of the first psychotic episode [3]. With each relapse, the functional recovery of patients to the pre-relapse level is difficult and shows a gradual decline [1, 4]. There are enormous medical costs and productivity losses associated with the relapse of schizophrenia. The treatment cost of recurrent patients is about 3 times that of non-recurrent patients [5, 6]. Prevention of relapse is a prerequisite for improving overall treatment outcomes in the management of schizophrenia [7].

Risk perception refers to patients’ feelings, awareness, and understanding of risk objects and risk characteristics [8]. Identifying the early signs of relapse and initiating timely treatment to reduce the probability of relapse and hospital admission are essential in the treatment plan of patients with schizophrenia [9]. According to the Health Belief Model [10], when patients can accurately perceive the likelihood and severity of the presence of health risks, they are likely to adopt appropriate behaviours for prevention. In a systematic review summarizing the results of 11 prospective studies, the identification of early risk of relapse in patients with schizophrenia ranged from 10% to 80% [11]. The reasons for the large disparity in the accuracy of early identification of the risk of relapse in patients must be explained, and the accuracy must be improved further. Aycock et al. [12] stated that overestimation of the risk of developing the disease predisposes individuals to a state of chronic stress and fear of relapse [13]. In contrast, underestimation might cause individuals to ignore the risk and affect their motivation and ability to cope. Hence, it is important to clarify the level and characteristics of risk perception of relapse in patients with schizophrenia.

Most of the current domestic and international studies have focused on the factors associated with relapse [14], relapse risk prediction [15], and measures for the prevention of relapse in patients with schizophrenia [9]. There are few qualitative studies that have explored the feelings associated with relapse [16]and persistent negative emotional experiences such as fear of relapse [13]. There is only one study in the field of psychiatric disorders on the perception of symptoms in patients with anxiety and depression; however, this study focused on the perception of the medical risk of the disease, which is different from the perception of the risk of disease relapse [17]. During the process of disease treatment and recovery, patients are often exposed to various risks. Different risk perception characteristics and levels might influence patients' medication behaviour and decisions [18]. Considering this, there are significant differences in disease cognition and medication behaviour among different patients with schizophrenia. This study used qualitative research for an in-depth exploration of the perception of risk of relapse among patients of schizophrenia with first-episode, first relapse, and multiple relapses and further analysed its dynamic characteristics. The results will provide theoretical support for the prevention of the relapse of schizophrenia and provide a basis for related nursing care.

## Methods

### Design

In this study, semi-structured qualitative interviews were used to interview Chinese inpatients and outpatients with schizophrenia. To explore their cognitive experience of their own risk perception of relapse, Colaizzi’s phenomenological seven-step method was used to analyze the data [19]. This study was designed and reported and followed the consolidated criteria for reporting qualitative studies COREQ checklist [20].

### Participants

In this study, we used purposive sampling to select participants. Patients with first-episode, first relapse, and multiple relapses of schizophrenic episodes were selected from outpatients and inpatients of psychiatric hospitals in Daqing and Chifeng from July 2022 to September 2022. Currently, there is a lack of consensus-based criteria for defining the first episode, first relapse, and multiple relapses of schizophrenic episodes. Therefore, the above concepts are operationally defined in this study. We combined the theme of this study and the definition by Xiao et al. [21] of relapse (change of antipsychotic medication; increased number of hospital visits; rehospitalization; close supervision due to self-harm, aggressive behavior, and/or suicidal or homicidal ideation. Patients who met at least one of these four criteria were identified as relapsing). At the same time, in order to ensure that patients have a complete experience of disease and relapse, all patients are required to have at least one hospitalization experience. Operationalization is defined as follows: The first episode refers to clinical remission after treatment of the first episode; The first relapse refers to the occurrence of at least one of the above four relapse criteria after the first remission; Multiple relapses refer to the occurrence of at least one of the above four relapse criteria after the first relapse and remission. The inclusion and exclusion criteria for the study are shown in Table 1. Participants were allowed to participate in the interview alone or accompanied by their family members, and no participant in this study requested a family member to accompany them. Therefore, all interviews were conducted with patients alone.

**Table 1:** Inclusion and exclusion criteria of participants

Inclusion criteria:
1. Age between 18 and 65 years.
2. Diagnosed by a psychiatrist using the International Classification of Diseases-10/Diagnostic and Statistical Manual of Mental Disorders-5 criteria.
3. It is consistent with the definition of first onset, first relapse, and multiple relapses defined in the study.
4. The disease has reached a stable stage: change in the total score on the Positive and Negative Syndrome Scale (PANSS) to less than 15, and the total score of less than 60 in the last 4 weeks.
5. Cognition is intact or slightly impaired.
6. The ability to provide informed consent
Exclusion criteria:
1. Informed consent is hampered by cognitive impairment.
2. Severe medical conditions and psychiatric disorders coexist.

### Data collection

The researchers had received specific training to conduct interviews and have the skills to conduct qualitative research. In this study, semi-structured face-to-face interviews were conducted to collect data. The interview questionnaire was prepared based on the research purpose and related literature. The interview questionnaire was revised after consulting experts in psychiatry, nursing, psychology, and other fields. Two patients who met the inclusion criteria were selected for the pre-interview (results of the pre-interviews were not included in the study analysis). The interview questionnaire is shown in Table 2. The questions were identical for all participants, and the researcher could adjust the order of the questions in the interview questionnaire as necessary to ensure the continuity of the interview. The researcher was introduced to the patient by the matron or the doctor-in-charge before the interview to alleviate the patient’s concerns. The interview settings were independent, quiet, and private, where participants could feel free to express their experiences. During the interview, the patient’s non-verbal characteristics (such as actions, expressions, etc.) were recorded, and interview techniques were used to maximize access to information. At the same time, attention was paid to the adverse emotional reactions of patients, such as crying. Researchers should immediately stop the interview or change the topic and give comfort. In this study, two participants were interviewed twice (one patient interrupted the interview for treatment and one patient interrupted the interview due to a family visit; both patients completed the entire interview during the second attempt). The rest of the participants were interviewed once. The duration of each interview was 15 to 30 min. Interview notes and reflective notes were taken during and immediately after each interview. We discontinued when the analysis reached data saturation: the interview has not added any new information to the data collection process [22].

**Table 2:** Outline of the interview

1. Do you know what disease you have?
2. What do you think about the relapse of schizophrenia?
3. How did you learn about the relapse of schizophrenia?
4. What factors do you think caused the relapse?
5. Do you know the signals before the relapse?
6. Do you think the disease will recur in the future?
7. If the disease relapses, what is the impact you are most worried about?
8. What have you done to prevent a relapse?
9. What are you planning to do in the future to prevent relapse?

### Data analysis

Within 24 hours of the interview's conclusion, the verbatim transcriptions of the audio recordings were reviewed. Data analysis was conducted independently by two experienced researchers using Colaizzi’s phenomenological seven-step method to extract themes [19]. (1) The audio recording equipment was used to record every interview, which was then transcribed. Every transcript was read several times and researchers highlighted the important points. (2) Re-read, highlight and extract significant statements of views and experiences directly related to the perception of relapse risk in patients. (3) Create definitions for all important assertions. After consultation, the two researchers came to an agreement. (4) Identify the developed meanings and group them into theme clusters. (5) Describe the investigated phenomenon exhaustively. (6) The essential structures of the perspective and experience of relapse risk in patients were described. (7) Visit the participants once again for confirmation. Researchers addressed any discrepancies until a solution was found. All participants provided their phone numbers and agreed to be contacted again.

### Trustworthiness

The credibility of the study was improved in 4 ways: trustworthiness, dependability, confirmability, and convertibility [23]. Respondents were recommended by their supervising physicians to participate in this study voluntarily, and the interviewers established a trusting relationship with the respondents before the interviews. Only patients who were willing to share their feelings were recruited to ensure the objectivity of the study results. Two researchers (YH, SYJ) analysed the data coding and theme and discussed the differences with ZYQ, a professor of mental health nursing, to enhance the reliability of data analysis. All audio recordings and textual data in the study were maintained so that they could be accessed if required later; the researchers themselves remained neutral throughout the study and reviewed their personal biases at any time. After completion of the data analysis, the textual data were returned to the respondents for verification to ensure the stability of the results.

### Ethical considerations

Ethical approval was obtained from the Ethics Committee of Harbin Medical University (HMUDQ20221110001), and it conformed to the ethical guidelines of the Helsinki Declaration. The content and purpose of this study were explained to the study subjects before the interview to ensure the confidentiality of the interview. The names of interviewees were replaced by numbers (P1-P22). Informed consent and permission to record were obtained from all participants. Written informed consent and permission to record were received from all participants.

## Results

### Description of the sample

Finally, 22 participants were included in this study, and no participants refused or quit, including 6 with first onset (P1, P13, P18, P20, P21, P22), 7 with first relapse (P4, P5, P10, P12, P15, P16, P19), and 9 with multiple relapses (P2, P3, P6, P7, P8, P9, P11, P14, P17). Other information is shown in Table 3.

**Table 3:** Sociodemographic of patients with schizophrenia included in the study

**Characteristics**	**Schizophrenia**
**Age range (years)**	18–64
**Source**	
Outpatient clinic	7
Hospitalization	15
**Sex**	
Male	16
Female	6
**Education level**	
Primary school or below	8
Secondary school	3
University	11
**Employment**	
Employed	9
**Family history**	3

### Perceptions of relapse risk and behavioural changes

**Fig. 1:**
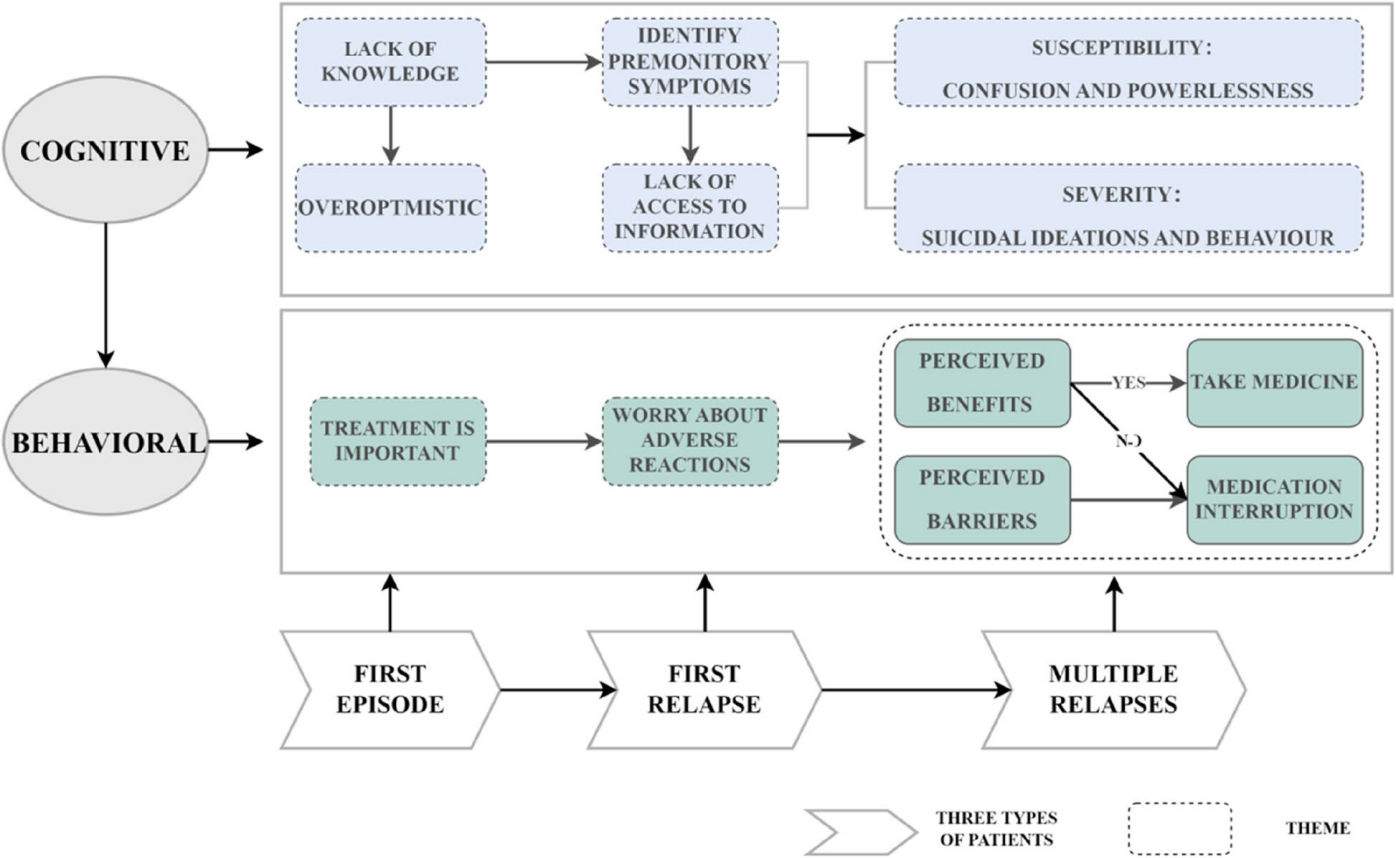
Frame diagram of risk perception of relapse in schizophrenia

We obtained nine themes that correspond to three types of patients. We discovered that in patients , the cognition of the risk of disease relapse and the corresponding behavioural response were dynamic. *At the patient's cognitive level:* Because first-episode patients lack understanding of the disease, relapse estimates are overly optimistic. Patients experiencing their first relapse have a preliminary understanding of the early warning signs of relapse, but there are no systematic or accurate channels for obtaining disease-related information. Patients who have had multiple relapses have a more comprehensive understanding of the disease and can demonstrate that it is easy to relapse and can have serious consequences. As a result, they experience confusion and powerlessness in disease control, and they may even engage in suicidal ideation and behaviour. *At the behavioural level of the patient:* Patients on their first episode are vaguely aware that treatment is critical. Patients experiencing their first relapse recognized the importance of treatment but became concerned about the drug's side effects. Patients with multiple relapses exhibit erratic medication behaviour that oscillates between perceived benefits and treatment obstacles, potentially worsening the disease (See Fig. 1).

### Perceived risk of relapse in patients who experienced the first episode of schizophrenia

Patients experiencing the disease for the first time lacked relevant experience and understanding of the disease, which led to some delay in the first treatment. Because of the lack of awareness of the disease, patients are too optimistic about the risk of relapse in the future, resulting in the inability to prepare for relapse in time. Most of them have a limited understanding of the importance of treatment, but this understanding is relatively vague and lacks of estimation of the difficulties that may exist in treatment.

#### Lack of knowledge about disease recognition and medical treatment

Since patients in the acute phase generally lack awareness about the disease, most patients with the first episode of the illness are found to be in an abnormal state by family members or people around them.

P01: “This is the first time I have had this disease. I didn't realize it. My family realized that I was sick. ”

Even if another part of the patients and their families were able to identify the disease state, some of them failed to seek timely consultation at psychiatric hospitals due to the lack of relevant knowledge and experience.

P18: "I am just upset for more than a month. I do not know which hospital to go to. ” P22: "I have not recovered after taking traditional Chinese medicine for more than twenty days. My sister's relative was treated here. Hence, I came here."

#### Overoptimistic estimation of the risk of relapse

Patients with first-episode lacked knowledge related to disease relapse or even motivation to understand the disease.

P21: "I do not know, and I do not want to know. " P22: "Then who knows? Sometimes I think I might be alright."

Some patients' knowledge of disease relapse is based on their own experience and self-inference. The understanding of relapse is vague and one-sided. Overall, there is underestimation or even over-optimism about the risk of relapse.

P1: "I think there is a risk of relapse for any disease in general, but I feel that my chances of relapse are very small.” P13: “I know that the disease may relapse. But if there are no big ups and downs, it may not relapse easily.”

Some patients even completely ignore the risk of disease relapse after stabilization, equating the control of symptoms with the cure of the disease and thinking that they will not relapse.

P18: "After taking medicine, the symptoms were relieved. So, there will be no relapse if it is cured. I am very confident."

#### Perceived importance of treatment

Antipsychotic medication is the primary treatment for schizophrenia. Patients with first-episode can initially realize the importance of medication for the treatment of the disease.

P1: "My mom said she will get more medicines prescribed from the hospital when necessary. I think taking medicine should also have some effect." When asked about the duration of the medication, the patient replied: "I guess it will take a few months. "

When asked whether they knew about the difficulties they might encounter during treatment and the adverse effects of medication, most patients said: "I have no idea" or "I didn't think about it." Only one patient searched for possible problems during medication through the online platform but knew little about it.

P21: "I have also searched through Baidu (a search engine), and I have seen the kind of people who do not eat after feeling better for a long time. I can be sure that I have not recovered yet, so I do not have this kind of idea."

### Perceptions of relapse risk among patients of schizophrenia with first recurrence

Patients who had relapsed had some understanding of the disease, especially the risk related to relapse and could further understand the risk of relapse and related information through their own experience and in other ways. However, the information obtained was not comprehensive or accurate. Patients still recognized the importance of treatment but had concerns about the adverse effects of treatment.

#### Initial awareness of relapse warning symptoms

A proportion of patients experiencing relapse for the first time were not yet able to identify the precursor symptoms of relapse very accurately.

P4: "I really do not know much about the early warning signs of relapse. " P5: "I do not know; there are no warning signs."

Because of the similarities between relapse symptoms and first-episode symptoms, some patients are able to determine whether they are developing a relapse.

P15: "The symptoms of the two attacks were similar, with the occurrence of auditory hallucinations." P19: "Similar to the first episode, I also had a headache, pain, and talking to myself. "

There is heterogeneity in the early warning symptoms of different patients. However, following the first relapse, the outlook of family members has changed from being confused and overwhelmed to be able to identify early warning symptoms more quickly and accurately and to seek timely medical treatment.

P4: "I am not aware of my illness, but sometimes I feel that my behaviour is out of my control. Then my family assumed I might have a relapse, and they sent me to this hospital. "

This can make up for the inability to correctly identify relapse during the onset stage due to the lack of insight.

#### Lack of systematic and accurate access to disease information

The patients said that the main sources to obtain disease information include the experience of people around them, psychiatrists, hospital publicity, and information on the network platform. There are many complicated information channels, and patients have a sense of uncertainty about the accuracy of information.

P15: "I heard my uncle said that stopping medication may lead to relapse (scratching her head, being confused)." P19: "When I get out of the hospital, I will check on the Internet. There are doctors on the Internet. It should be accurate, right (doubtful)?

Moreover, even if patients with a first relapse obtain information about the disease through some sources, their overall awareness is still inadequate. They were unaware of what information they should seek.

P12: "I did not know anything about it. However, a few days ago, I read the bulletin board on the wall of your hospital. After I read it, I understood it. This disease should be a mental illness, but that is all I know. Is there anything else I need to know?"

It is worth noting that through the interviews, some patients realized their lack of knowledge about the disease and were motivated to learn about it.

P4: "No one ever told me about the disease. Mostly, I did not ask. I really should know about it. Next time I meet my doctor, I will ask her."

#### Perception of drug withdrawal is related to relapse

Patients who have experienced relapse can conclude the relationship between drug withdrawal and disease relapse.

P4: “If you do not take medicine, you do not control it if you sometimes have strange ideas. But when taking medicine, I consciously control these thoughts.

The main reason for drug discontinuation is adverse effects of medication.

P15: "The gynaecologist said that maybe this medicine had some effect on my menstruation, so I stopped. This relapse may have something to do with the withdrawal of drugs at that time.

Most of the patients affirmed that they would comply with medication in the future and initially recognized that relapse could lead to worsening of the disease; however, patients are worried about the adverse effects of antipsychotics.

P15: "I think taking medicine has a bad effect on my body. The key problem is that not taking medicine has a greater impact because I am afraid of relapse. P19: "Nearly ten tablets have several side effects. I do not understand how to solve so many adverse effects (anxious, confused).

### Perceived risk of relapse among patients with multiple relapses of schizophrenia

Most patients with recurrent understand that relapse is common and can have serious consequences. As a result, patients experience confusion and powerlessness, as well as suicidal thoughts and behaviours. Patients' medication behaviour oscillates between perceived benefits and treatment barriers. It may even worsen the disease as a result of this process.

#### Susceptibility to relapse: confusion and powerlessness

After many relapses, most patients believe that the disease is easy to relapse. Recurrent episodes of the disease lead to progressive exacerbation, and patients develop a sense of confusion and powerlessness about preventing disease relapse.

P2: "I believe I am prone to relapse. I have had it 6 or 7 times. I cannot recall anything specifically. Sometimes I feel like I have been sealed by heaven. I cannot seem to get rid of this disease."

Some patients believe that the disease cannot be cured, the disease relapse cannot be prevented, and they even lose confidence in disease treatment.

P7: "Anyway, it has relapsed many times. There is no radical cure, so we will make do with it. If I can figure it out, I will think of it. If I cannot figure it out, there is nothing I can do."

#### Severity of relapse: suicidal thoughts and behaviour

In a state of illness, patients show behaviours such as beating and scolding family members, which brings great harm to their families. At the same time, there is a greater demand for family members to care for them following an illness. As a result, the disease relapse has a significant impact on the patients' families.

P2: "The serious impact is that the family is unhappy. It is hard to trouble my sister, and so is my wife. "

In addition, the alienation and discrimination of the people around the patients lead to a sense of stigma.

P9: "It is just that a lot of people know I am sick. And then I felt like everyone was talking about me behind my back. I do not think it is good for me."

After the patients return to society, adapting and integrating into social life is another difficult challenge in the rehabilitation process. When patients are unable to adapt to social life, they might even have suicidal thoughts.

P14: "I do not want to socialize with people, and I talk less. I just cannot adapt to society. In this society, I want to escape and even have suicidal thoughts."P2: "I am not interested in playing chess, reading, or learning. Interest has decreased, and my temper has grown. I did not want to live anymore. I killed myself." "

#### Effects of perceived benefits and barriers on medication behaviour

Perceived benefit is a critical motivator for patients to change their behaviour [24, 25]. When patients believe that the treatment will effectively reduce the consequences of the disease, they will actively pursue it; when there is no obvious benefit, they will not pursue it. Some patients in this study are unable to perceive the benefits of treatment or believe that treatment is ineffective. They are sceptical of the treatment's efficacy.

P2: "Taking medicine does not work at all. When you are in a bad mood, it is useless for you to take any medicine. If you have a relapse when you take medicine, it will not work even you take a lot of medicine. I just do not think the drugs are working."

Some of the patients who have experienced multiple relapses realize that drug treatment is an important measure to prevent a relapse. They stated that they would continue to take medication in the future:

P17: "I have been taking the medication prescribed by Director Wang for nearly a year. I no longer feel bad. I am no longer sick. If I do not take the medication, I will not relapse in one day, but I will relapse in two days."

However, if the patient encounters difficulties and resistance to the medication behaviour, the individual may abandon the behaviour [26]. Due to the many adverse effects of medication, it causes considerable agony to patients. The patient swayed between taking or not taking medicine, leading to further aggravation of the disease.

P3: "I am taking medicine in the hospital, but I will not take it when I am discharged from the hospital. Just because taking medicine makes me feel bad, it is like a nervous breakdown. " P6: “I believe I should also take medication. But taking medicine is unpleasant and exhausting, so I refuse to take it.”

## Discussion

Schizophrenia is a serious mental illness with a high rate of relapse and disability. Perceivetion and identification of early warning signs of relapse is a cornerstone of relapse prevention within mental health services [27]. Consistent with previous studies, there are a series of problems in the perception of the risk of relapse and the behaviour of preventing relapse in patients with schizophrenia [28, 29]. At the cognitive level, patients lack not only disease-specific knowledge and relapse risk awareness, but also the relevant disease information pathways. At the behavioural level, patients change their medication behaviour with some regularity, and overall medication compliance is generally low.

In the interviews, we found that although the perception of the risk of disease relapse among patients could increase with the progression of the disease, the overall risk of relapse is ignored and underestimated. Previous studies have shown that there is a common problem of low disease awareness in patients [30]. Patients cannot adopt an appropriate behaviour pattern if they cannot master the relevant knowledge of their own disease [31]. This leads to the inability to identify early signs of relapse in time and take effective measures for prevention. One patient in this study summarized the reasons for relapse as follows: People are not so aware of this disease. It is really just not that well recognized. Under the guidance of the Health Belief Model [10], providing accurate disease-related information early on is necessary to enhance patients' awareness of the benefits of preventive health behaviours for relapse. Strengthening communication and educational efforts on relapse risks by healthcare professionals may be an effective approach to addressing this issue [32]. Therefore, this study suggests that healthcare professionals should educate patients to help patients perceive their own risk of relapse [33]. They can also create a platform for support and mutual assistance for patients [34]. By sharing their knowledge and direct experience with other patients with the same disease and treatment experience, awareness about the disease can be increased [35]. This helps build the right attitude toward treatment and helps patients who are not aware of the risk of relapse.

In this study, patients used various sources to obtain disease information. Among them, there are many uncertainties, especially through the experience of the people around them and the information obtained from online platforms. It has been reported that when patients with schizophrenia search for disease information online, they are not always sure which information can be trusted [36]. Among all the sources of information, that from medical institutions is the most reliable [37]; however, patients expressed in the interviews that "doctors are too busy" and “there are too many outpatients to talk to the doctor", so it is difficult to contact doctors and get a detailed explanation. This is consistent with previous findings that individuals with schizophrenia often do not receive professional help [38]. However, patients still need access to disease information and the ability to seek medical services [39]. Therefore, it is necessary to explore a channel that can provide systematic and accurate information to patients anytime and anywhere [40]. However, the digital health management model based on the Internet and mobile platform can be used as an easily accessible and low-threshold source of information [41]. Therefore, hospitals can create specialized digital management platforms based on big data technology to provide scientific and accurate information to patients in need anytime and anywhere [42]. A study highlighted that there is an imbalance between the transmission and reception of information in doctor-patient communication, and online communication should particularly address this problem [43]. Therefore, when communicating with patients , the message should be made easy to understand and accept. We should regularly test the scientific nature of the information obtained by patients and the correct acceptance rate of information.

Our study found that the treatment attitudes and behaviours in patients are dynamic. It is necessary to dynamically evaluate, provide feedback, and manage them according to their different characteristics. Currently, antipsychotic medications are the first-line treatment for schizophrenia, according to current guidelines [44]. A meta-analysis including 35 studies that reported a pooled estimate of medication non-adherence found that the non-adherence rates in schizophrenia, major depression, and bipolar disorder were 56%, 50%, and 44%, respectively [28]. Schizophrenia is a lifelong illness, and non-adherence to medication is common and widespread [45]. In a systematic review [46], it will be necessary to identify the patient’s perception of medication, illness, and behaviour when taking medication in order to determine the next intervention that will be appropriate based on the patient’s needs to improve adherence. Based on the results of this study, we identified the characteristics of dynamic changes in patients' attitudes towards medication . In the future, according to the major challenges and needs of patients in different stages of the disease, targeted intervention strategies can be formulated to improve medication compliance.

Special attention should be paid to the treatment of newly diagnosed patients. Standardized drug treatment during the first episode is helpful in reducing relapse and improving long-term outcomes [47]. While in clinical practice, first-episode patients often experience dropout during treatment [48, 49]. Patients with first-episode understand the importance of medication but are unaware of the complications that may arise during treatment. Non-adherence to treatment is frequently observed, with the most common factor leading to relapse being medication discontinuation, as indicated by a 5-year follow-up study on first-episode schizophrenia [50]. Therefore, the primary goal of first-time patient management is to assist patients in identifying potential problems during treatment and teaching relevant coping skills. Implementing ongoing care after the first onset of illness, early identification of prodromal symptoms, and personalized management are also crucial aspects [51].

In this study, the impact of drug adverse effects on first-relapse patients began to emerge, and it became an increasingly significant issue for patients to continue taking drugs. These findings are consistent with previous studies [49, 52], indicating that as medication dosage increases and the occurrence of side effects rises, and the likelihood of treatment non-adherence by patients also increases. This suggests that patients should be highly cautious about the risk of self-discontinuation or dosage reduction if they experience medication side effects. In addition, individuals vary considerably in their risk of side effects and how these effects are experienced. Risk-benefit assessments about whether to prescribe antipsychotic medication for an individual should be made according to specific drugs and the specific situation (i.e., actual benefits and harms expected or experienced by an individual).

In patients who have experienced multiple relapses, the benefits and obstacles of perceived treatment are the main reasons that affect medication adherence. This is consistent with previous research. The majority of patients are aware of the preventive effects of antipsychotic medication on relapse, which is a primary reason for their adherence to medication [53, 54]. However, the occurrence of numerous adverse reactions due to long-term medication is a major barrier to adherence [55, 56]. Therefore, it is necessary to implement measures to optimize the management strategies for patients on long-term medication. These findings suggest that individuals requiring long-term medication should undergo regular follow-up assessments in order to streamline their medication regimen and address the issue of complex medication regimens, thereby enhancing adherence among patients [57]. In order to improve medication adherence among patients, healthcare professionals should encourage patient involvement in shared decision making with their physician to express their preference and opinion in treatment selection to increase their adherence to medication [58]. Otherwise, long-acting antipsychotic injections can be administered to assist oral medication to decrease relapse [59].

### Limitations

There are some limitations to this study. First, as with all qualitative studies, the demographics of the research team, such as sex, education level, and occupation, might potentially influence the interpretation of the interviews and data. Second, participants were recruited from only 2 regions in China, and thus the results cannot be generalized. Third, family members of patients were not included in this study. However, family members play a very important role in patients' risk perception of relapse and might be the "first persons” to perceive the risk of relapse. Therefore, the perception of the risk of relapse by the family members of patients with schizophrenia is a direction for future research. Fourth, we were unable to obtain information about the perception of relapse in the same set of patients over time. In the future, we could consider longitudinal qualitative research to obtain a more comprehensive and in-depth understanding of the experience or behaviour of individuals or groups over time [60].

## Conclusions

Patients of schizophrenia with first onset, first relapse, and multiple relapses have different views about the risk of relapse, attitude towards treatment, and medication behaviour. They lack timely, accurate, and systematic access to relapse-related information. They neglect and underestimate the risk of disease relapse. In the absence of an accurate perception of the risk of relapse, patients cannot take appropriate actions to prevent a relapse. Therefore, it is strongly recommended that healthcare workers explore a health management plan in line with the dynamic development of the disease according to the number of relapses among patients.

## Data Availability

Due to the privacy of the participants involved in the study data, the datasets generated and/or analyzed in the study are not currently publicly available but are available from the corresponding authors of this study upon reasonable request.
